# Parental experiences of caring for children who have learning disabilities and procedural anxiety in hospital: An interpretive phenomenological study

**DOI:** 10.1111/cch.12990

**Published:** 2022-03-01

**Authors:** Lauren Murdoch, Yan‐Shing Chang

**Affiliations:** ^1^ Florence Nightingale Faculty of Nursing, Midwifery and Palliative Care King's College London London UK

**Keywords:** complex needs, learning disability, paediatrics, parents, procedural anxiety, qualitative research methods

## Abstract

**Background:**

Children with learning disabilities (LD) are more likely to have health conditions that require hospital attendance than children without LD. Like all children, they can experience fear and distress related to procedural anxiety. Parents play a key role in managing procedural anxiety in children with LD. No previous published qualitative studies have explored parental experiences of caring for a child with LD and procedural anxiety in hospital.

**Objectives:**

To explore how parents experienced caring for their child with LD and procedural anxiety in hospital.

**Methods:**

A purposive sample of six participants were recruited through a Facebook group for parents of children with LD. Remote semi‐structured interviews were conducted via telephone, Microsoft Teams or Whatsapp. Interviews were transcribed verbatim and analysed using interpretative phenomenological analysis.

**Results:**

Five key themes were generated: (1) *Emotional toll*: parents characterized their experiences as highly emotional; reporting feeling stressed, anxious and worried. (2) *Restraint and holding*: parents spoke of their experiences of restraint which was largely viewed as negative and sometimes inappropriate. (3) *Advocacy*: parents articulated their responsibility as advocates for their children. (4) *Going it alone*: parents were extremely proactive in managing their child's anxieties but some also felt highly‐pressurized and isolated. (5) *Inconsistency and uncertainty*: parents experienced inconsistency and uncertainty in their children's care from healthcare professionals which led to anxiety and frustration.

**Conclusion:**

Parents of children with both LD and procedural anxiety experienced many challenges. Parents' expertise must be utilized by clinicians when caring for children with LD and procedural anxiety whilst ensuring appropriate support for parents. Nurses require specific training in psychosocial interventions to enhance care for children with LD and procedural anxiety. Further research identifying effective nursing strategies to enhance parental experiences would be beneficial to improve care to this patient group.

**Key messages**

- Caring for a child with LD and procedural anxiety in hospital is a challenging experience for their parents.
- Parents were extremely proactive in supporting of their children, yet some felt over‐relied upon by healthcare professionals. Nurses need to recognize and value parents' expertise whilst ensuring adequate support for parents themselves.
- Specific guidance is needed in relation to holding and restraint of children with LD who have procedural anxiety to ensure safe and compassionate care.
- That staff lack skills required to care for children with LD and procedural anxiety is congruent with wider criticisms of healthcare inequalities affecting children with LD.

## 1 INTRODUCTION

*Learning disabilities* (LD) is the preferred term used by the UK National Health Service (Department of Health, 2001) to describe reduced intellectual ability and difficulty with everyday activities. Internationally, the term ‘intellectual disability’ is used with the same purpose. Approximately 2.5% of the children aged 0–17 years in the United Kingdom are living with a LD. This represents over 351,000 children, 118,000 of which are aged 0–5 years (Office for National Statistics, 2019). Causes of LD in children are diverse, encompassing congenital conditions, genetic causes, and acquired brain injury from accident or epilepsy; approximately 43% of children with LD have unexplained causes (Courtman & Mumby, 2008). The number of children with complex needs (including LD) is growing due to advancements in healthcare technology increasing survival from acute and chronic childhood illness (Pinney, 2017). Compared with children without LD, children with LD are more likely to require healthcare interventions due to coexisting health problems, ranging from dental procedures to neurosurgery (Courtman & Mumby, 2008). They may also find it more difficult to tolerate the sensory stimulation, regimes and unfamiliar social contact associated with hospital settings (Short & Calder, 2013).

Procedural anxiety refers to fear, anxiety and worry experienced when undergoing a medical or surgical procedure. Often this can be linked to previous negative experiences (e.g., needle‐phobia) whereby negative cycles of fear and pain lead to heightened distress (Noel et al., 2012). Anxiety is known to exacerbate perceptions of pain and is associated with prolonged recovery periods, increased need for analgesia, nausea and vomiting (McCann & Kain, 2001), as well as dysfunctional behaviour changes post‐operatively, for example, tantrums and bed‐wetting (Kain et al., 1996).

Procedural anxiety may cause such distress that procedures are postponed, abandoned or necessitate sedation or restraint (Short & Calder, 2013). This poses additional risks to the child including side effects from medication, accidental injury or delayed treatment. These interventions can be traumatic and may further entrench procedural anxiety. All children have the right to healthcare under the United Nation's *Convention on the Rights of the Child* (United Nations, 1989), and children with LD are rightfully entitled to equity in their healthcare outcomes (Government Equalities Office, 2010). Children with LD are already susceptible to poorer quality of care than their peers (Mimmo et al., 2020), and poor care for procedural anxiety may further entrench existing health inequities for these children.

The study is underpinned by the family‐centred care model, which is widely accepted as the paediatric standard‐of‐care by clinicians across healthcare settings (Kuo et al., 2012). This model acknowledges parents as experts in their children and emphasizes the need to engage with them collaboratively, in partnership with healthcare professionals (HCPs). Family‐centred care also recognizes that a child exists within the family unit and that children's well‐being is inherently linked to that of their immediate family (Gilson et al., 2018). Therefore, it is important to consider parental experiences in nursing practice. This is increasingly highlighted in contemporary literature, which calls for the need for healthcare services to partner with parents and carers and enable children with LD to voice their experiences of care (Mimmo et al., 2020). Although limited previous published qualitative studies have investigated the topic of parental experiences of caring for a child with LD in hospital (Avis & Reardon, 2008; Bates et al., 2019; Brown & Guvenir, 2009; Taghizadeh et al., 2019), none have yet addressed parental experiences of caring for a child with both LD and procedural anxiety in hospital. This study addresses this gap.

## 2 METHODS

This study utilized interpretative phenomenological analysis (IPA). The essence of IPA is to capture the complexity of an individual's lived experience (Pietkiewicz & Smith, 2014), which is appropriate for this study exploring the lived experiences of parents who cared for their child with LD and procedural anxiety in hospital. The research question was: how do parents experience caring for their child with learning disabilities and procedural anxiety in hospital?

### 2.1 Participant recruitment and sampling

Potential participants were approached on a Facebook group for parents of children with LD via a recruitment advert. All initial correspondence between the researcher and potential participants was by email. The study design targeted six participants who were sampled via purposive sampling (see Table 1). The sample size was typically small in line with IPA methodology, favouring depth over breadth (Moser & Korstjens, 2017). This is intrinsic to IPA's main concern of giving each participant's discourse full appreciation while acknowledging its limitations for generalizability (Pietkiewicz & Smith, 2014).

**TABLE 1:** Inclusion and exclusion criteria

Inclusion criteria	Exclusion criteria
A parent of a child with LD and procedural anxiety aged 0–18 whom they have prior experience of caring for during a hospital visit in the United Kingdom	Parents with learning disabilities.
Competent and willing to either have a telephone call, use Whatsapp (to instant messaging, video or voice call), or use Microsoft teams (to voice or video call) for interviews.	Parents whose hospital experience have occurred outside the United Kingdom
Willing to talk about their experiences of caring for their child during an episode of procedural anxiety	

### 2.2 Data collection

Remote one‐to‐one semi‐structured interviews were conducted between August 2020 and September 2020 at a time chosen by participants and conducted via their choice of Whatsapp (instant messaging), telephone or Microsoft Teams. The participants were encouraged to find a quiet, private space of their choosing. The author interviewed the participants from home. No non‐participants were visibly present with the exception of the husband and child of one participant (it is unknown if other household members were present for Whatsapp or telephone interviews).

The interviews followed a schedule (see Appendix A) designed and piloted for this research. No modifications were made to the schedule following the pilot. For telephone and Microsoft Teams interviews, audio recordings were taken via dictaphone and transcribed verbatim. Some field notes were made immediately after the interviews.

### 2.3 Data analysis

Data were analysed as per the IPA process described by Smith and Osborn (2003), generating the key themes and subthemes of the interviews through a cyclical process incorporating multiple readings and making notes, transforming notes into emerging subthemes, seeking relationships and clustering themes (see Table 5). Both authors analysed the data and agreed on the final themes and subthemes. Data saturation is not a conventional goal of IPA (Smith, 2004); instead, data adequacy (Vasileiou et al., 2018) is considered a more suitable concept in IPA as individual experiences are so unique and complex that data can never be truly saturated. As analysis of interview data generated consistent themes across transcripts, the sample size (*n* = 6) and data collected from this study were judged to be adequate. Findings were returned to participants for comment; no participants provided feedback.

### 2.4 Ethics

Ethical approval was granted by the King’s College London research ethics committee (LRU‐19/20‐19986). Informed consent was received from all participants. Participants were assured of confidentiality and anonymity, but were also aware that confidentiality could not be maintained if a disclosure was made that could raise concerns regarding the well‐being and safety of themselves or their child.

### 2.5 Reflexivity

The first author is a paediatric nurse working full‐time clinically in a large tertiary paediatric hospital in central London and is experienced in caring for children with complex needs and disabilities from an acute medical and surgical setting and in the community. Being familiar with the patient group, the researcher's background may have affected her expectations of the experiences participants would report. The first author conducted interviews and identified herself to participants as a nurse which may have affected the dynamic of their interaction. Participants were not known to the authors prior to study commencement. The second author is a health service researcher with expertise in qualitative research and provided objective views during the research project including the data analysis and interpretation process.

## 3 FINDINGS

### 3.1 Participants' characteristics

Of a total of 21 individuals who expressed an interest in participating, six participants provided informed consent. No reasons were specified by those who chose not to participate. The participant demographic information is displayed in Table 2. All the participants were female although the participant requirement aimed at both female and male. There might be a range of reasons for this. For example mothers typically assume primary caregiver roles due to a varied range of complex social factors, not limited to social norms and additional financial burden associated with caring for a child with LD in the United Kingdom (Buckner & Yeandle, 2017), or an overrepresentation of women recruited via Facebook for healthcare research (Whittaker et al., 2017).

**TABLE 2:** Participants' demographics

	Number of participants (total *n* = 6)
Gender	
Female	6
Male	‐
Age of participant (years)	
35–40	2
41–45	3
46–50	‐
51–55	‐
56–60	1
Region (UK)	
South East England	4
North East England	2
Age of child with LD (years)	
>3	‐
3–6	2
7–9	1
10–12	‐
13–15	2
16–18	1
Number of siblings	
Only child	1
1	1
2	2
3	2

Table 3 displays each child's primary diagnosis, procedures associated with anxiety and interview format. Children in this demographic are typically complex with multiple co‐morbidities but only primary diagnoses have been displayed in Table 3 to avoid compromising participant anonymity.

**TABLE 3:** Child's primary diagnosis, procedural anxiety and interview format

	Participant (pseudonym)					
	Jane	Suzie	Heidi	Lydia	Maxine	Sophie
Child's primary diagnoses disclosed in interview	Epilepsy, autism spectrum disorder	*Undisclosed*	Trisomy 21	Noonan's syndrome, autism spectrum disorder	Trisomy 21	Trisomy 21
Procedures described in interview	Nasogastric tube placement, blood test	Blood test	Blood test	Echocardiogram, impedance study, imaging, blood tests	Blood test, dental treatment	Blood test
Interview format	Whatsapp instant messaging	Whatsapp instant messaging	Whatsapp instant messaging	Microsoft teams	Telephone	Telephone

### 3.2 Interview characteristics

The average duration and transcript word count for each format of interview are shown in Table 4. Telephone interviews had the shortest average duration (32 minutes), while interviews via Whatsapp instant messaging yielded the shortest average transcript word count (2338 words). In the context of IPA, this is a shorter duration than expected for an interview and could be due to participants' conflicting demands on their time; several participants mentioned their time was limited by caring for their child. Parents spoke remarkably concisely regarding their experiences and perhaps had prepared what they wanted to share. Whatsapp's credibility as an interview tool remains mixed in methodological literature, although it should be acknowledged that some academics argue nonverbal communication typically emerges in different forms in instant messaging scenarios (emoticons, capitalization, phatic statements, etc.) and does not necessarily lack the complexity of spoken words (Lijadi & Schalkwyk, 2015).

**TABLE 4:** Interview characteristics

Type of interview	Number of interviews conducted	Average duration of interview (minutes)		Average word count of transcript (total number of words)	
		Mean	Range	Mean	Range
Whatsapp (instant messaging)	3	81	63–91	2338	1812–3310
Telephone	2	32	33–31	4227	3170–5283
Microsoft Teams	1	41	41	3550	3550

### 3.3 Themes

Five key themes were generated from the data. Table 5 presents themes and examples of additional supporting quotations.

**TABLE 5:** Themes, subthemes, notes and quotations

Theme	Example of emerging subthemes	Notes	Example of supporting quotations
Emotional toll	Dread and anticipation	Frequency of appointments; child's behaviour can be challenging; strong negative emotions; anxiety‐inducing for parents.	*We do actually dread every single hospital appointment, just because you do not know how he's going to be […] We dread even taking him to the GP‐ and, you know, he has nearly every aspect of his medical and developmental side being monitored, so the appointments are endless*. (Lydia)
	Burden of maintaining a ‘brave‐face’	Hiding emotions from child; aware that child will be affected by parental distress; emotionally challenging.	*I was feeling emotional but trying not to show that emotion because I did not want my child to get more distressed … so it's really hard …* (Maxine)
Restraint and holding	Awareness of long‐term impact on child	Concerns for the future; fear; negative view of restraint.	*I know it scares me that if it continues and he gets bigger and stronger that they will have to restrain him manually and I'm extremely against that and it will cause his fears to become so much worse*. (Heidi)
	Fears child will feel betrayed by parent	Afraid to damage child's trust in parent; concerned for relationship; feeling conflicted; challenging.	*It's about our relationship as well is not it? You do not want to damage your relationship with your child, but they are thinking ‘my mum knows and she's still putting me through this procedure’, so it's really hard*. (Lydia)
	Undermined trust in clinicians	Following clinician's instructions; regret; parent education; perceived lack of clinician expertise regarding autism; feeling guilty for child's prior bad experiences.	*I feel responsible as well because we physically done that [*sic*], not the doctors, not the nurses, that was us holding him down because we thought that was what we had to do and now obviously we have done courses and stuff on autism and that's the worst thing to do … And I thought they would have known that*. (Lydia)
Advocacy	Parents acutely aware of their role as child's advocate	Be the advocate. Be helpful.	*I like to be the advocate for my son and to tell staff he's a superhero. It helps him, and we all like to be helpful. (Heidi)*
	Feelings from being child's advocate	Intense emotions; fear; hysteria; feeling out of control; not being listened to; frustrated; guilt.	*I was near on hysterical at one point. I was so frightened for her. The trauma I knew she was going through, no one would listen to me, no one would help her, that's how it felt‐ that I am her voice and I am being silenced. I felt like, and still do, that I let her down*. (Jane)
Going it alone	Parents independently and proactively initiating interventions for their child	Parents travelling weekly to take child to hospital; parents initiating intervention; time and energy consuming; highly committed to child's well‐being.	*We did it on a Wednesday because we did not go to nursery on a Wednesday […] getting the train from here to [the hospital] and back again takes most of the day really […] but it is quite tiring, it is your whole day which is then gone, but if it helps him‐ being in hospital‐ it also helps us I suppose, it's worth it in the long run*. (Lydia)
	Feeling let down by healthcare professionals unable to meet highly complex and specific needs of child	Seeking help; feeling isolated; healthcare professionals unable to help child; let down by the ‘experts’; expectation that the healthcare professionals will be able to help.	*It's awful, you turn to people for help and guidance and they are meant to be the experts. It makes you feel very alone. (*Jane)
Inconsistency and uncertainty	Availability of staff inconsistent	Routine appointments varied in staffing; specialist staff not reliably available.	*We always go to the same hospital, the same unit, the same place, but one year you'll go and get a play specialist, two extra staff and the nurse, type‐thing, other years I've been and they only have the one nurse in there*. (Sophie)
	Uncertainty during the COVID‐19 pandemic	Concerned about visiting arrangement during COVID; impact of COVID	*I've had many sleepless nights worrying about this over COVID if he was admitted and me not being allowed, or me then presenting with symptoms*. (Suzie)

#### 3.3.1 Emotional toll

When asked to characterize their experiences of caring for their child in hospitals, all participants spoke explicitly and unambiguously about the emotional toll of caring for their child when they were acutely distressed. Five parents expressed acute feelings of regret or guilt, when describing a traumatic incident that had happened to their child during a procedure.

For me it's exhausting, worrying, guilt over Harry. And stressful. The build up to going is stressful too […] overall it is very very upsetting. (Suzie)

We just felt like we were torturing him. (Lydia)

Three parents spoke of disguising their emotions, choosing to put on a ‘brave‐face’ for their children, and the difficulty of maintaining this at a time when they themselves were upset. The emotions identified by parents were almost always negative. However, one parent also reported a more hopeful feeling of anticipation that her son might tolerate the procedure well, which would ultimately be of benefit to him.

#### 3.3.2 Restraint and holding

All parents talked about being asked to hold or restrain their child. Parents' experiences ranged from therapeutic holding to unplanned restraint. They described doing both with great reluctance and discomfort and cited this as one of the most distressing aspects of caring for a child during a procedure.

I laid on the bed with her on top of me, screaming her heart out whilst I held her head, other nurses held her arms and legs whilst the tube was put in. (Jane)

Parents were also highly aware of the long‐term effects of negative experiences on their child.

Several participants referred to feelings of betrayal when restraining their children for procedures. They were concerned that their child could not understand the necessity of the treatment and were afraid of damaging their child's trust in them. Three parents disclosed conflicting emotions as they felt restraint was the kindest approach to take with their children, even though this was distressing for them both, as their anxiety was so severe that there was no other way the procedure could be performed. Suzie described feeling cautious of how this would be received by staff, even though she felt this was in her son's best interest as multiple attempts to elicit cooperation from him were futile and ultimately ended in further distress.

My son […] usually takes up to 5 people including myself to hold him. I'm always worried they will not agree to continue due to his distress, which would mean going back again (Suzie)

Lydia described feeling pressured to restrain her child, and her subsequent regret, as retrospectively she believed the restraint was an unhelpful and inappropriate intervention for her son. This experience appears to have damaged her trust in their healthcare providers.

#### 3.3.3 Advocacy

Parents reported mixed experiences of advocating for their child. Often the circumstances of advocating was to request different HCPs be allocated, or to challenge care plans.

From that point onwards I've insisted that someone who takes blood is someone who's really proficient […] and can do it quickly, in and out, one attempt. (Maxine)

Parents' reported advocating for their child was met with mixed reactions. Two parents felt their concerns were dismissed and had to ‘prove’ their child could not tolerate a procedure before alternatives were considered; they felt they were pressured into attempting an intervention they anticipated would not be successful, which was upsetting and frustrating.

Three parents also acknowledged that their child was likely to remain under the care of specialists for some time and seemed to feel conflicted about advocating for their child. Although they were mindful of being perceived as ‘obstructive’, their child's well‐being was their upmost priority. They were also aware of the overarching financial aspects of their child's care.

It's hard because they have got a job to do, you know Simon needs the procedures and the scans and stuff, and it is so frequent as well, so we understand. And we understand there's financial implications and stuff like that as well. (Lydia)

Two parents reported that they felt HCPs were quick to assume their child's level of ability, based on their initial presentation, which was often misinterpreted and caused upset and frustration for parents. In these circumstances, parents were quick to correct HCPs.

Just because a child is twelve with Down syndrome does not mean she likes Tinky‐Winky from the Teletubbies. (Sophie)

Two parents discussed gaining confidence advocating for their child over time, particularly after experiencing poor care from HCPs, and ‘learning the hard way’. Parents recognized the limitations of doctor's expertise within the context of rare and complex diseases and the individual needs of their child.

I've found with my daughters' care you kind of have to be an expert in everything because things change so much and new research comes out […] so you kind of have to be an advocate and do that, and of course every child is different. (Maxine)

#### 3.3.4 Going it alone

Participants described feeling isolated, unsupported or left to ‘fend for themselves’ in several aspects of the lived experience while independently taking the lead on their child's care. Parents were considered ‘alone’ in the sense that they had taken on the roles, unique to their child's needs, that HCPs would not expect of parents of other, less complex, children. The notion of ‘alone’ was also conceptualized in the sense that parents of children with unique and highly specific needs were often the only people able to provide care for their child, to the extent that two mothers reported not feeling supported by fathers in this respect. Participants acknowledged an added weight of responsibility that they could not simply hand over care to nurses or friends and family.

I'm not sure how he would cope if I had to leave him. (Suzie)

Parents reported instances where it seemed they had been exceptionally proactive and independent in trying new strategies to ease their child's distress. For example, Lydia described taking her child into hospital on a weekly basis to de‐sensitize them to the hospital environment. She spoke positively of this experience as it allowed her son to begin to tolerate being inside the hospital entrance without casing distress, despite being a very time‐consuming process. Parents referenced information they had read about in academic literature, discussed specialist courses they had taken, recommended books, shared petitions and discussed contemporary hot‐topics. Heidi described how her decisions around restraint were influenced by other parents' experiences she had observed on online forums, rather than clinicians' advice.

I always told them not to restrain him, so they never did. I know of other parents on the forums who write things like ‘I cannot hold him down because he's too strong now’ and I did not want to be in that position. (Heidi)

Jane described how she was told by a HCP that they had ‘never met a child like [daughter] before’. She described her disappointment and frustration when being told that they did not know how to help her child. These experiences appeared to be inherent to the nature of parenting a child with rare, complex or highly specific needs.

#### 3.3.5 Inconsistency and uncertainty

Inconsistency and uncertainty were demonstrated in parents' discussions about HCPs, parents' decision‐making, and the ongoing COVID‐19 pandemic. All parents spoke of inconsistency in relation to their interactions with HCPs in terms of skills, competencies and availability of specialist staff. Some parents expressed having reservations leaving their child in the care of nurses, based on their child's specific needs associated with their LD which staff were unable to meet.

As he's non‐verbal, staff would have to know Makaton and even knowing it they would struggle to understand the way he signs. (Suzie)

Though parents reported negative experiences throughout the majority of the interviews, all parents described at least one positive interaction they had had with HCPs. They spoke highly of these interactions and highlighted how valuable these experiences were.

The first time, when there was a specialist, the nurse went so far as to draw blood out of their own arm to demonstrate what was going to happen. (Heidi)

Parents also experienced uncertainty fundamentally linked to the complex nature of their child's needs. One parent reported her child's behaviour was typically unpredictable, which itself was anxiety‐inducing. Another mother reported her child's additional needs made decision‐making more challenging. Two parents spoke of their anxieties surrounding the current COVID‐19 pandemic disrupting their routine care which added to the burden of uncertainty.

It's [COVID‐19] just setting us back I think. The little progress we have made it just feels like we are having to go back to square one and try all over again. (Lydia)

## 4 DISCUSSION

To the best of our knowledge, this is the first qualitative study to explore parental experiences of caring for a child with both LD and procedural anxiety in a hospital setting. A key finding from this study was the theme of ‘going it alone’. This theme is partially reflected in the existing literature on the topic of parental experiences of caring for children with LD in hospital. For example, Avis and Reardon (2008) identified the pressure put on parents caring for their child with LD in hospital in terms of the delegation and delivery of care with parents reporting often feeling over‐relied upon by HCPs. However, their evidence did not demonstrate the full extent to which parents can feel isolated as the sole providers of their child's care. Neither did it identify the extent to which parents proactively and independently initiated interventions to improve their child's experience when their child also had procedural anxiety, for example, bringing their child to hospital regularly to desensitize them. This has implications for family‐centred care as HCPs need to utilize parents' invaluable expertise, whilst also recognizing the need to provide support and optimize parental well‐being.

The findings highlighted the emotional toll experienced by participants in the study. Participants cited intense emotions associated with their child's procedural anxiety characterizing their feelings as stress, anxiety and guilt. These parents may benefit from additional emotional support. This could be delivered in many forms, such as incorporating parents' needs in care planning on admission, or a formal referral to psychological services. However, there are minimal studies on the effectiveness of nursing interventions to support parents' mental health in parents of children with LD, and many nurses report lacking confidence and skills discussing mental health with parents (Gilson et al., 2018).

Although important, provision of extra emotional support alone could be viewed as treating the symptom not the cause. Parents will inevitably experience many stresses and anxieties which are specific to caring for children with LD and exacerbated by procedural anxiety; these may be alleviated simply from improved patient care. Findings from Oulton et al. (2020) suggest ‘getting it right’ from the onset is vital in establishing parent trust in the healthcare system. Various types of psychosocial intervention have been found to be successful in reducing child anxiety and pain and improves procedural success but are often overlooked by clinicians (Chrisler et al., 2021). Many of these interventions could be carried out within the scope of nurses' roles, for example preparing patients with video, social story or medical play, teaching patients coping strategies such as breathing techniques or procedural support such as distraction. However, these interventions have not been fully evaluated from a cost–benefit perspective (Chrisler et al., 2021) and may not be feasible for widespread implementation considering workforce pressures such as nursing shortages.

One of the most concerning findings from this study was of the use of restraint and holding, which was a major concern for participants. Bates et al. (2019) also reported similar findings from parents of children with cleft palette and LD. The improper restraint of people with LD is an enduring issue attracting widespread attention in recent years with the Whorlton Hall (2019) and Winterbourne View (2011) hospitals scandals in England. These bought mainstream attention to systemic abuse towards adults with LD within NHS institutions. Some academics continued to criticize an inadequate response from the government (Richards, 2020). Similarly, the forthcoming Oliver McGowan mandatory training (Department of Health and Social Care [DHSC], 2019) highlights enduring concerns of staff not valuing parent's knowledge and expertise.

There remains a distinct lack of clarity and unity with regard to clinical guidelines for children with LD (Bray et al., 2019). While recent guidelines, *Reducing the Need for Restraint and Restrictive Intervention*, (DHSC & Department of Education, 2019) have been published, they are not specific to healthcare settings. Although other guidelines exist for holding in paediatrics (Royal College of Nursing, 2019), these do not address the needs of children with LD. National evidence‐based guidelines for restraint in children with LD in healthcare settings, with consideration to procedural anxiety, are essential to the safety and well‐being of child patients. However, this would require a strong, comprehensive evidence base which is not yet available. Further research in this area is highly desirable.

Parents were concerned about the skills and knowledge of HCPs caring for their child with LD. This has also been highlighted in previous studies (Avis & Reardon, 2008; Brown & Guvenir, 2009; Sharkey et al., 2014), with many parents reporting feeling unable to leave their children with ward staff. This sentiment is mirrored by nurses themselves in a recent study in which nurses reported feeling less confident and capable caring for children with LD than those without (Oulton et al., 2018). Although Mencap's *Getting It Right* charter (2010) calls for all paediatric units to have access to a dedicated LD nurse to support staff, Oulton et al. (2019) suggest they are not effectively or efficiently used. Similarly, parents have reported inconsistent access to specialist staff including specialist liaison nurses (Brown & Guvenir, 2009) and play specialists (Taghizadeh et al., 2019). The value of play specialists in preparation and distraction of children is well‐evidenced, although provision is not reliably available or utilized effectively (Gulyurtlu et al., 2020).

The Equality Act (Government Equalities Office, 2010) sets out the legal duty of all healthcare services to consider the needs of all children, young people and adults with LD, who are entitled to expect quality in the outcomes of their healthcare provision. This involves making reasonable adjustments to prevent disadvantage arising from any disability that could negatively impact a child. More recently, *The Learning Disability Improvements Standards for NHS Trusts* (NHS, 2018) outlines the rights of patients to receive safe and personalized care with the same quality and outcomes of their peers. Given the numerous concerns reported by parents, it seems HCPs are missing opportunities to make reasonable adjustments and failing to meet these standards. This is similar to recent findings from Oulton et al. (2015) that young people with LD reported the ‘little things’ which comprised individualized care were of upmost importance to patients. Oulton et al. (2015) asserted that developing partnerships with parents was pivotal in the delivery of care.

A seminal 2017 review by the National Council for Disabled Children which asserted that ‘at a fundamental level the skills needed for working with our group of children did not seem to be fully recognised, articulated or appropriately valued’ (Lenehan, 2017, p. 28). These inequalities are further intersected by other factors, for example, social‐economic status (Public Health England, 2015). The issue is complex and warrants further enquiry and engagement with children, families and the wider community to improve the experiences of children with LD across all sectors. Lenehan noted that services were reliant on the ‘particular skills, interests and determination of the clinicians involved’ (Lenehan, 2017, p. 18). This resonates with the ‘ad hoc’ positive experiences of participants reported in this study. Given the extent of wider workforce challenges, such as COVID‐19 and the global nursing shortage which is an urgent and multifactorial issue in contemporary nursing (Marufu et al., 2021), implementing widespread training may be challenging. Nonetheless, this is vital to ensuring equitable health outcomes for all children.

This study is limited by the IPA methodology as data are restricted to experiences of few individual participants and lacks generalizability. Further research is required to substantiate the findings of this research. It would also be desirable to capture the experiences of fathers and ethnic minority groups, given that healthcare inequalities are intersected by many demographic factors (Public Health England, 2015). Using Whatsapp for conducting interviews also potentially limits the findings as current methodological literature is mixed on the credibility of its use. Using digital technologies ensured the transmission of viruses was minimized during the COVID‐19 pandemic while it was important that participants were familiar with the interface. Due to the limitations of these platforms, children with LD were not included as participants as these platforms typically have age‐restrictions which posed additional ethical concerns and may not be an appropriate or effective form of communication for individuals with communication barriers. This would affect the quality of the data collected. However, further research to capture children with LD's experiences using accessible methods is needed as Oulton et al. (2017) assert it is vital to challenge the notion that it is acceptable to exclude this population of patients from research on the basis of their inability to participate.

## 5 CONCLUSION

Parents characterized their experiences as highly emotional reporting stress, anxiety and worry. Appropriate support should be offered to parents, ranging from informal support provided by nursing staff as part of their care plan to comprehensive, formal support from specialist clinicians. Parents articulated their responsibility as an advocate for their child and were extremely proactive in managing their child's anxieties, but some also felt highly‐pressurized and isolated. Parents' expertise must be utilized when caring for their child with LD and procedural anxiety; but they must not be over‐relied upon and provided with appropriate support as per family‐centred care.

Parents discussed their experiences of restraint which was largely viewed as negative and sometimes inappropriate. Given the lack of guidance relating to holding and restraint in children with LD and procedural anxiety, further high quality research in this area is needed to build clinical guidance to support safer care for parents. Parents experienced inconsistency and uncertainty in their care, even when visiting the same ward, which was a source of anxiety and frustration. Nurses require more specific training in the care of children with LD and procedural anxiety, this could include a range of psychosocial interventions such as preparation, procedural support and coping strategies. Further methodological research relating to use of Whatsapp instant messaging as a platform for interviews would be beneficial, as well as strategies to recruit fathers and those from diverse backgrounds to future research on this topic.

## CONFLICT OF INTEREST

The authors declared no conflict of interest.

## ETHICS STATEMENT

Ethical approval was granted by the King's College London research ethics committee (reference: LRU‐19/20‐19986).

## PATIENT CONSENT STATEMENT

Written informed consent was received from all participants prior to all data collection.

## Data Availability

Full transcripts of the data are not available as participants in this study did not agree for their transcripts to be shared publicly.

## APPENDIX A INTERVIEW SCHEDULE

### Semi‐Structured Interview Guide

Beginning of Interview:

Welcome and introduce myself.

Reiterate the aims of the project. Confirm what is meant by procedural anxiety.

Remind people of key issues‐ data will be kept confidential and anonymous, unless there is a reason researcher needs to act, participants can feel free to withdraw at any time.

Demographic questions:

What is the participant's age?

What is the participants gender?

What is the age of child with learning disability?

Do they have any other children, and their ages if applicable?

What region of the country does the family lives in?

Interview Guide

1. What has been your experience of caring for your child with a learning disability, whilst they are having procedural anxiety?
2. Can you describe how this experience made you feel?
3. What stands out to you the most about being with your child during a procedure?
4. What support have you received from staff? Do you feel this is the right level of support?
5. How do you think your child's experience have affected your experiences, if at all?

Interview expected to last approx. 45–60 minutes via video or voice call.

End of Interview

Thank participants for time.

Offer Support Services sheet.

Ensure they have researcher's details if they have any further questions.

Remind of deadline to withdraw consent.

Confirm whether they would like a copy of the finished report when it is available and how they would be happy for researchers to contact them with this.
